# Severe respiratory changes at end stage in a FUS-induced disease state in adult rats

**DOI:** 10.1186/s12868-016-0304-5

**Published:** 2016-10-28

**Authors:** Kasey L. Jackson, Hemangini A. Dhaibar, Robert D. Dayton, Sergio G. Cananzi, William G. Mayhan, Edward Glasscock, Ronald L. Klein

**Affiliations:** 1 Department of Pharmacology, Toxicology, and Neuroscience, Louisiana State University Health Sciences Center, 1501 Kings Hwy, Shreveport, LA 71130 USA; 2 Department of Cellular Biology and Anatomy, Louisiana State University Health Sciences Center, 1501 Kings Hwy, Shreveport, LA 71130 USA

**Keywords:** Amyotrophic lateral sclerosis, Adeno-associated virus, FUS, Gene therapy, Respiration, TDP-43

## Abstract

**Background:**

Fused in sarcoma (FUS) is an RNA-binding protein associated with the neurodegenerative diseases amyotrophic lateral sclerosis (ALS) and frontotemporal lobar degeneration. ALS manifests in patients as a progressive paralysis which leads to respiratory dysfunction and failure, the primary cause of death in ALS. We expressed human FUS in rats to determine if FUS would induce ALS relevant respiratory changes to serve as an early stage disease indicator. The FUS expression was initiated in adult rats by way of an intravenously administered adeno-associated virus vector serotype 9 (AAV9) providing an adult onset model.

**Results:**

The rats developed progressive motor impairments observed as early as 2–3 weeks post gene transfer. Respiratory abnormalities manifested 4–7 weeks post gene transfer including increased respiratory frequency and decreased tidal volume. Rats with breathing abnormalities also had arterial blood acidosis. Similar detailed plethysmographic changes were found in adult rats injected with AAV9 TDP-43. FUS gene transfer to adult rats yielded a consistent pre-clinical model with relevant motor paralysis in the early to middle stages and respiratory dysfunction at the end stage. Both FUS and TDP-43 yielded a similar consistent disease state.

**Conclusions:**

This modeling method yields disease relevant motor and respiratory changes in adult rats. The reproducibility of the data supports the use of this method to study other disease related genes and their combinations as well as a platform for disease modifying interventional strategies.

## Background

Amyotrophic lateral sclerosis (ALS) is a neurodegenerative disease affecting upper and lower motor neurons. The disease causes a progressive paralysis including paralysis of the diaphragm and other respiratory muscles. Respiratory complications and failure are the most common cause of death in ALS [1, 2]. Prior to respiratory failure, ALS patients exhibit breathing abnormalities including hypercapnia [partial pressure of carbon dioxide (*p*CO_2_) >40 mmHg] and hypoxemia [partial pressure of oxygen (*p*O_2_) <80 mmHg] [3, 4] as well as increased respiratory frequency and decreased tidal volume [5]. Disease relevant respiratory abnormalities have been demonstrated in a mutant form of copper, zinc superoxide dismutase 1 (mSOD1) mouse model [6], and a rat model of mSOD1 exhibited decreased motor output from the phrenic nerve [7, 8]. In humans, a variety of genetic etiologies can result in ALS and here we studied breathing in a model based on the RNA-binding protein fused in sarcoma/translocated in liposarcoma (FUS). We studied breathing parameters with a sensitive plethysmography system in a rat model. The main goal was to study breathing changes induced by FUS in an effort to establish a sensitive early disease physiological marker for detecting either the efficacy of therapeutic intervention or potentiation of the disease state by disease modulators.

FUS is an RNA- and DNA-binding protein that regulates transcription, alternative splicing, and mRNA transport [9]. FUS shares homology and some overlap in function with the RNA-binding protein transactive response DNA-binding protein of 43 kDa (TDP-43). Both proteins are associated with ALS and the related disease frontotemporal lobar degeneration (FTLD) [10–20] in terms of causative mutations and neuropathology (proteinopathy). Wild-type FUS is found aggregated in FTLD with FUS-positive inclusions (FTLD-FUS) [17–19] and has also been found in pathological aggregates in sporadic ALS [21]. Mutations of other RNA-homeostasis proteins are also associated with ALS, including TATA-binding protein associated factor 15 (TAF15) [22, 23] and Ewing’s sarcoma breakpoint region 1 (EWSR1) [24], which further underscores the importance of RNA metabolism in underlying disease mechanisms. We have previously established rat models of progressive paralysis by expressing TDP-43 [25–27]. Here we expressed FUS in rats, in order to test the hypothesis that another ALS related RNA-binding protein would exert a similar disease state, presumably by interference of normal RNA metabolism. Overexpression of wild-type FUS has been shown to be toxic in yeast [28–30] and drosophila [31], and a variety of rodent models have been developed using either mutant or wild-type FUS, both forms showing evidence of motor impairments and ALS-like pathology [32–35]. We used a novel and unique viral vector expression system in adult rats to study respiratory changes. Rat physiological parameters such as heart rate and breathing are closer to humans than mice, so rats are therefore advantageous to study breathing parameters.

The adeno-associated virus (AAV) vector system is an advantageous method to control the onset of expression within specific neuronal populations. AAV9 can be delivered intravenously in rats to yield wide-scale expression in the central nervous system (CNS) including efficient transduction of spinal motor neurons necessary for motor performance [25]. When TDP-43 was expressed in this manner, a highly consistent motor phenotype developed over time [25]. The first study used neonatal rats which provide very efficient gene transfer [25]. However, we recently adapted the approach to adult subjects which are more relevant to adult onset diseases such as ALS and FTLD [26]. Here we administered adult rats with AAV9 FUS and studied motor and respiratory parameters over time, including analysis of blood pH and blood gas content. We tested if the motor and respiratory sequelae would arise concomitantly or if one sequela would precede the other. Plethysmography was also studied in AAV9 TDP-43 transduced rats for the first time to address if a similar phenotype was induced by the two disease-related RNA-binding proteins.

## Results

### FUS-induced disease state: decreased weight and motor impairments

**Fig. 1:**
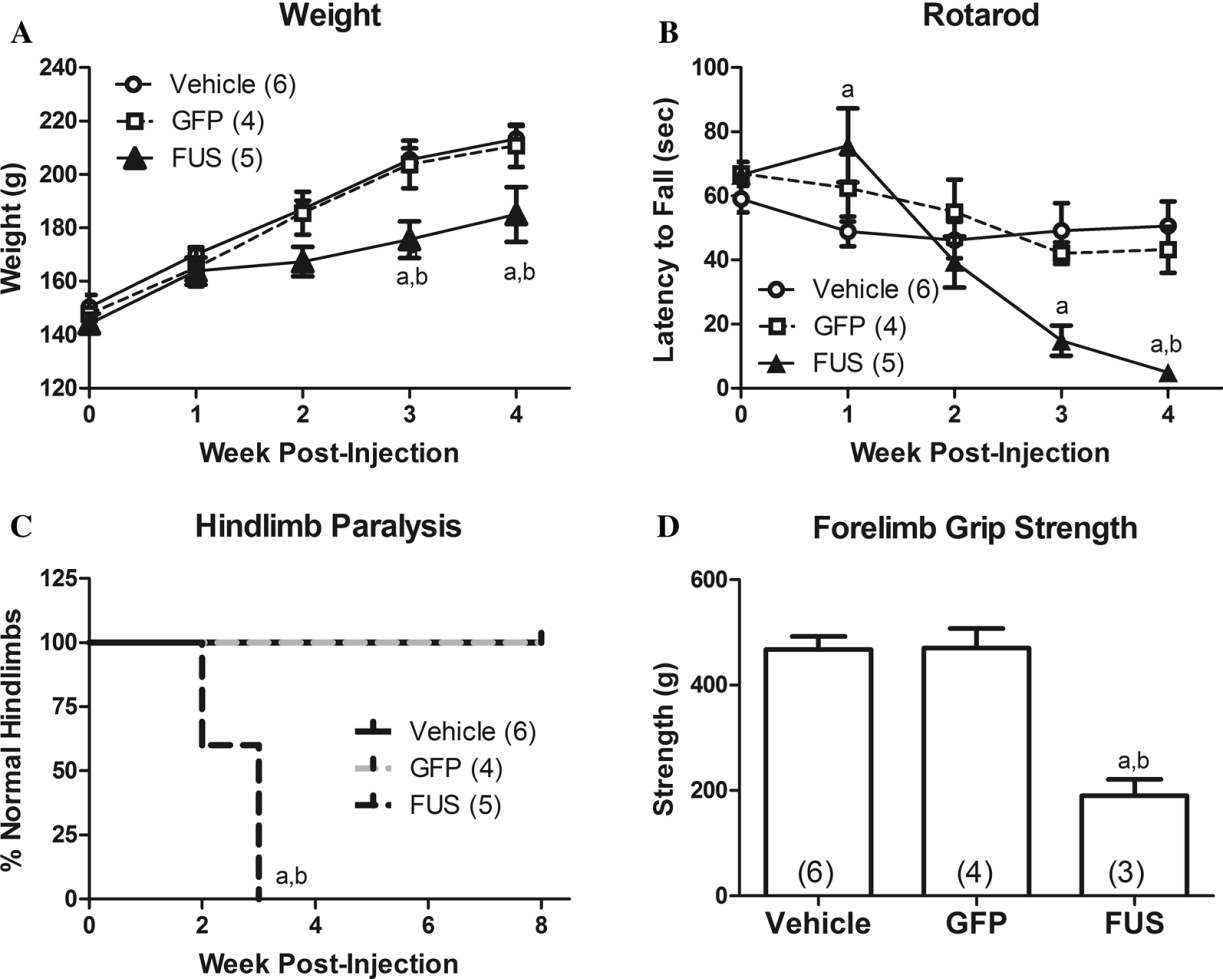
FUS gene transfer causes progressive motor impairments. **A** There was an indication of lowered weight gain in FUS rats by ANOVA (see “Results”). The FUS group weighed significantly less than the vehicle and GFP groups at 3 and 4 weeks post-injection (p < 0.05–0.01). **B** FUS rats were significantly impaired for rotarod performance by ANOVA with significant differences found at 1, 3, or 4 weeks compared to the vehicle group (p < 0.05–0.001) and at 4 weeks compared to the GFP group (p < 0.01). **C** All of the FUS rats developed hindlimb clenching during the escape reflex while no vehicle or GFP rats showed abnormal hindlimb clenching during the study (p < 0.01). **D** The FUS group had decreased forelimb strength at 4 weeks compared to both vehicle and GFP (p < 0.001). There were no significant differences between the vehicle and GFP groups in any of these measures. “a”, significant difference between FUS and vehicle groups; “b”, significant difference between FUS and GFP (ANOVA/Bonferroni post-tests in** A**,** B**, **D** and Mantel-Cox log rank analysis in **C**; n = 4–6/group as indicated)

To examine the effects of FUS gene transfer in adult rats, three groups were examined: (1) AAV9 FUS (human wild-type); (2) AAV9 green fluorescent protein (GFP); and (3) vehicle-only controls. The animals were weighed just before gene transfer and weekly up to 8 weeks (Fig. 1a). There was a significant effect of time on weight gain (two-way repeated measures analysis of variance (RM ANOVA), F_4,48_ = 232.20, p < 0.0001) as expected. There was a trend for an effect of treatment group (F_2,12_ = 12, p = 0.065), and there was a significant interaction between time post-injection and treatment group on weight (F_8,48_ = 7.58, p < 0.0001), suggesting some differences in weight gain between treatment groups over time. For example, at the time points of 3 and 4 weeks, the FUS group weighed less than the vehicle and GFP groups (Bonferroni post-test, p < 0.01 and 0.05, respectively).

To determine if rats administered AAV9 FUS developed motor impairments, animals were evaluated by rotarod, escape reflex, locomotor activity chamber, and forelimb grip strength. There were no differences found between the vehicle and GFP control groups on any of these measurements. On rotarod there was a significant effect of treatment group (two-way RM ANOVA, F_2,12_ = 4.36, p < 0.05) indicating that FUS affected rotarod ability. There was a significant effect of time on rotarod performance (F_4,48_ = 13.97, p < 0.0001), as we have previously observed in young to middle age rats [25]. There was a significant interaction between treatment group over time (F_8,48_ = 5.58, p < 0.0001) indicating that the FUS rats exhibited progressively impaired rotarod ability over this period. The FUS group showed significant rotarod deficits compared to the vehicle group at 3 and 4 weeks post-injection (Bonferroni post-test, p < 0.01 and 0.001, respectively) and at 4 weeks post-injection compared to GFP animals (Bonferroni post-test, p < 0.01; Fig. 1B). For the escape reflex, all of the vehicle and GFP animals retained hindlimb extension when lifted by the tail whereas all of the FUS animals developed an abnormal hindlimb clenching (Mantel-Cox log-rank test, p < 0.01; Fig. 1C). Hindlimb clasping is an indicator of a lesion in the motor pathway [36]. However, in the locomotor testing, there were no significant effects of FUS gene transfer on rearing or distance traveled in this study using this vector dose and interval (data not shown). Forelimb grip strength in the FUS group was significantly decreased compared to both vehicle and GFP animals at 4 weeks post-injection (analysis of variance (ANOVA)/Bonferroni, p < 0.001; Fig. 1D). These motor tests suggest that gross motor function and coordination (as measured by rotarod) in rats administered AAV9 FUS are impaired due to both dysfunctional hindlimbs (hindlimb clenching) and forelimbs (decreased forelimb grip strength).

### FUS-induced disease state: plethysmographic changes

**Fig. 2:**
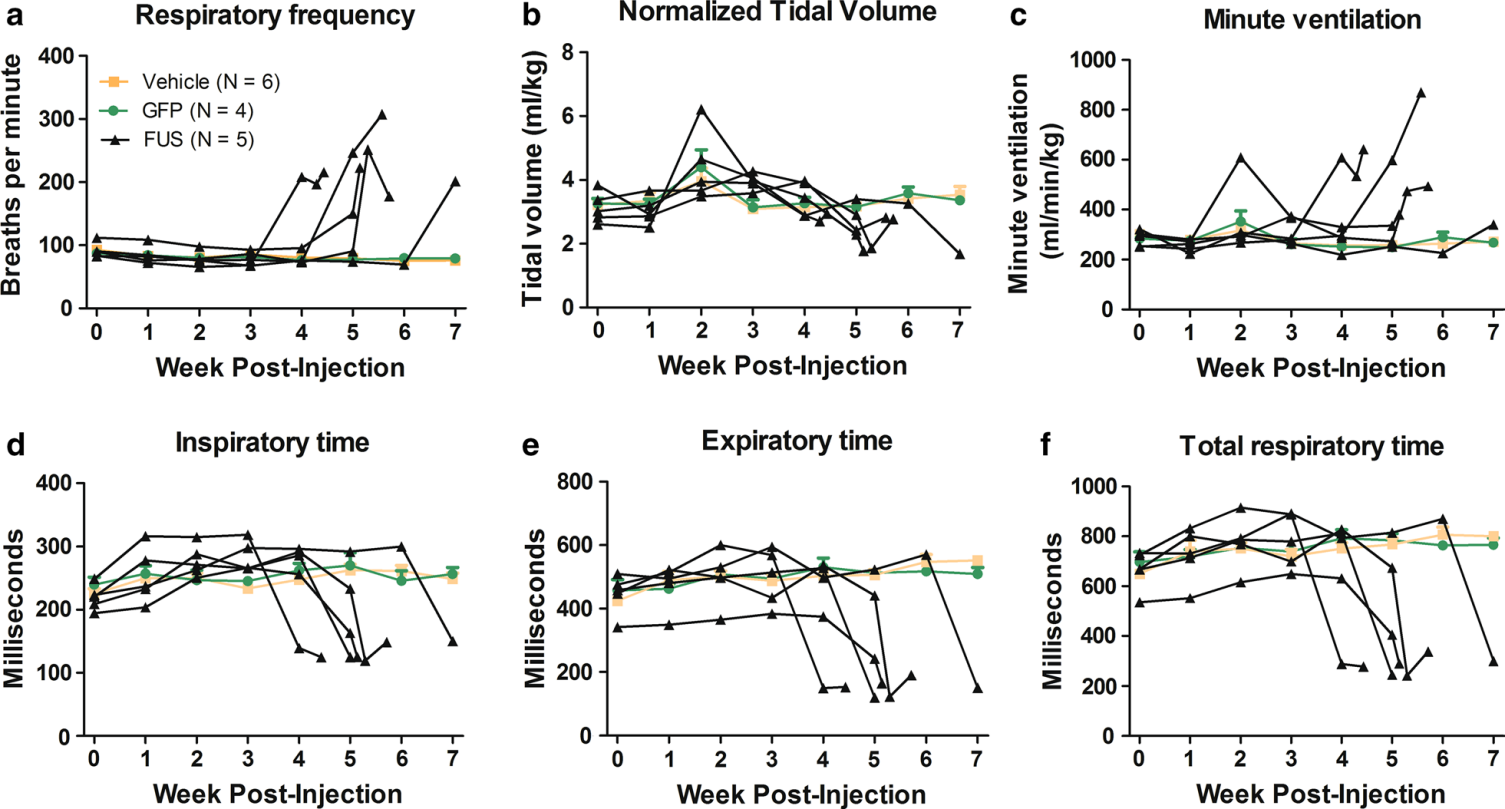
Changes in respiratory parameters in FUS rats over time. Respiratory parameters studied include respiratory frequency (**a**), tidal volume (**b**), minute ventilation (**c**), inspiratory time (**d**), expiratory time (**e**), and total respiratory time (**f**). Each FUS rat is displayed as a separate *black line*, and the average values ± SEM of vehicle and GFP animals are shown in *orange* and *green* respectively

**Fig. 3:**
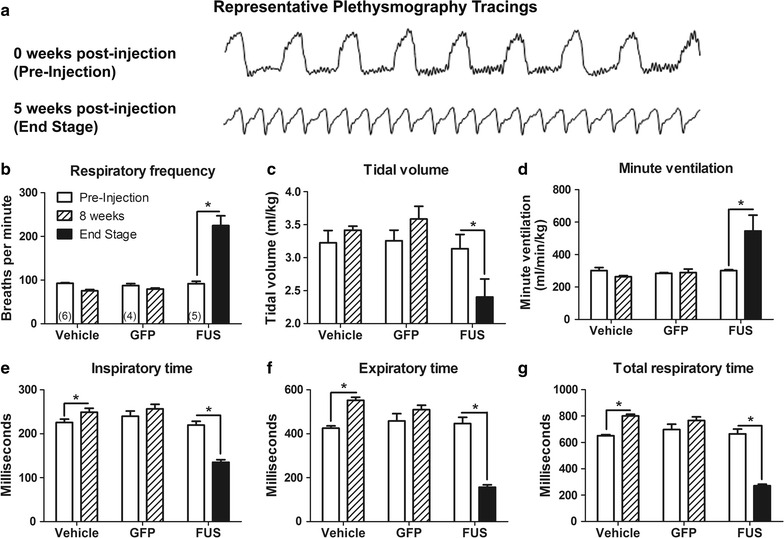
Rapid, shallow breathing in AAV9 FUS rats at end stage. **a** Representative plethysmography tracings of 5 s of respiration in a FUS rat at pre-injection and end stage (week 0 and week 5). **b** Respiratory frequency was increased in the FUS group at end stage (p < 0.001). **c** Tidal volume was decreased in FUS rats at end stage (p < 0.01). **d** Minute ventilation was significantly increased at end stage in FUS rats (p < 0.01). **e** The vehicle group showed increased inspiratory time over 8 weeks (p < 0.05); whereas, the FUS group showed decreased inspiratory time at end stage (p < 0.001). **f** Expiratory time was increased in the vehicle group after 8 weeks (p < 0.01) and decreased in the FUS group (p < 0.001). **g** Total respiratory time was increased in the vehicle group (p < 0.05) and decreased in the FUS group (p < 0.001). The n in **b**–**g** is 4–6/group as indicated in **b**. *Asterisk* indicates p < 0.05

Using unrestrained whole-body plethysmography, respiratory frequency, tidal volume, minute ventilation, inspiratory time, expiratory time, and total respiratory time were measured weekly throughout the study (Fig. 2). Between weeks 4 and 8 post-injection, FUS animals began to display progressive breathing impairments characterized by rapid, shallow breathing patterns (Fig. 2). The last plethysmography session before death or euthanasia was considered “end stage” which varied from 4 to 8 weeks depending on the individual animal treated with AAV9 FUS or AAV9 TDP-43. Due to this range of onset times, we compared the baseline to the end stage of disease progression in the FUS and TDP-43 animals. Vehicle and GFP control rats were not affected in this manner, so baseline was compared with 8 weeks post-injection in the controls. For respiratory frequency, there were significant effects of vector and time as well as an interaction between the two (two-way RM ANOVA, F_2,12_ = 38.48, p < 0.0001; F_1,12_ = 22.18, p < 0.001; F_2,12_ = 41.94, p < 0.0001, respectively). There was no difference in vehicle or GFP control rats between time points; however, FUS rats developed significantly increased respiratory frequency at end stage (Fig. 3b; two-way RM ANOVA/Bonferroni, p < 0.01). Tidal volume represents the lung volume of air displaced with normal inhalation and exhalation in the absence of any extra effort and is then normalized to body weight. There was a significant effect of vector as well as an interaction between vector and time (two-way RM ANOVA, F_2,12_ = 4.45, p < 0.05; F_2,12_ = 8.54, p < 0.005, respectively). There was no difference between pre-injection and 8 weeks in vehicle or GFP control rats, but tidal volume was significantly decreased at end stage in the FUS rats (Fig. 3c; two-way RM ANOVA/Bonferroni, p < 0.01). Minute ventilation is the volume of air displaced over one minute and is the product of respiratory frequency and tidal volume, normalized to body weight. There was a significant effect of vector, a trend for significance of time, and an interaction between vector and time (two-way RM ANOVA, F_2,12_ = 7.30, p < 0.01; F_1,12_ = 4.40, p = 0.058; F_2,12_ = 7.18, p < 0.01). Minute ventilation at end stage in FUS rats was increased (Fig. 3d, two-way RM ANOVA/Bonferroni, p = 0.01). For inspiratory time, there was a significant effect of vector and time as well as an interaction between the two (two-way RM ANOVA, F_2,12_ = 22.24, p < 0.0001; F_1,12_ = 10.45, p < 0.01; F_2,12_ = 61.35, p < 0.0001, respectively). For expiratory time, there was a significant effect of vector, a trend for significance of time, and an interaction between vector and time (two-way RM ANOVA, F_2,12_ = 75.85, p < 0.0001; F_1,12_ = 4.35, p = 0.059; F_2,12_ = 54.41, p < 0.0001, respectively). Total time, as expected, had significant effects of vector and time and an interaction between vector and time (two-way RM ANOVA, F_2,12_ = 88.61, p < 0.0001; F_1,12_ = 9.12, p < 0.05; F_2,12_ = 80.79, p < 0.0001). Inspiratory, expiratory, and total times all showed small but significant increases from pre-injection to 8 weeks for the vehicle group (Fig. 3e–g, two-way RM ANOVA/Bonferroni, p < 0.05, 0.01, 0.01, respectively). This could suggest acclimation to the testing chamber. Furthermore, it is not uncommon to observe normal changes in motor performance over time in controls [26]. However, this pattern was reversed in FUS rats which exhibited decreased inspiratory and expiratory time (Fig. 3e–g, two-way RM ANOVA/Bonferroni, p < 0.01 and 0.001, respectively), indicating impairment of normal respiration due to FUS. The breathing abnormalities at end stage were clearly visible in the home cage as rapid, pulsatile hyperventilation.

TDP-43, another RNA-binding protein implicated in ALS and RNA homeostasis, was tested for effects on plethysmography in a similar manner. Four animals were administered AAV9 TDP-43, which all developed similar breathing changes as the FUS group above. At end stage, measurements from TDP-43 animals indicated elevated respiratory frequency [233 ± 37 breaths per minute (bpm)], reduced tidal volume (1.86 ± 0.28 ml/kg), and increased minute ventilation (431 ± 55 ml/min/kg), decreased inspiratory time (152.6 ± 37.4 ms), decreased expiratory time (128.1 ± 18.9 ms), and decreased total respiratory time (280.1 ± 43.0 ms). We did not record pre-injection baseline for the TDP-43 rats. We compared the end stage values from the TDP-43 rats with the 8 week time point values from the two control groups (vehicle and GFP) by ANOVA/Bonferroni. The TDP-43 rats showed significantly increased respiratory frequency, reduced tidal volume, increased minute ventilation, and decreased inspiratory time, expiratory time, and total respiratory time compared to the controls (p < 0.05–0.0001). When comparing the TDP-43 group to the FUS group at the end stage, there was a similar amplitude of effect of FUS as TDP-43 gene transfer on respiration.

### Hypoxemia and acidosis accompany rapid breathing

Due to the tachypnea seen in diseased rats, arterial pH and blood gases were evaluated at end stage as a measure of cardiopulmonary function in a small subset of animals (Table 1). Though preliminary, there appeared to be similar changes in both the FUS and TDP-43 animals for the blood parameters measured. In the FUS and TDP-43 animals, blood pH was below 7.35, indicating acidosis, whereas none of the vehicle or GFP animals showed blood pH < 7.35. The blood pH of the FUS animals was significantly lower than the vehicle animals even with this small cohort size (ANOVA/Bonferroni, p < 0.05), and the blood pH of the TDP-43 animals was significantly lower than either the vehicle or GFP groups (p < 0.01). Some of the FUS and TDP-43 animals also exhibited abnormally low blood oxygen saturation (<90%). The FUS and TDP-43 animals had significantly lower oxygen saturation levels than either the vehicle or GFP groups (ANOVA/Bonferroni, p < 0.05, p < 0.001, respectively for both FUS and TDP-43). The FUS animals showed below normal *p*O_2_ (<70 mmHg) though not statistically significant, and the TDP-43 animals had statistically decreased *p*O_2_ (ANOVA/Bonferroni, p < 0.01). *p*CO_2_ appeared elevated relative to normal values (>50 mmHg) in some of the FUS and TDP-43 animals though these measurements were not statistically different than controls. In contrast to FUS and TDP-43, GFP gene transfer did not alter blood pH, oxygen saturation, or *p*O_2_ relative to the vehicle group.

**Table 1:** Arterial blood pH and gases

	GFP (n = 4)	Vehicle (n = 6)	FUS 1	FUS 2	TDP-43 1	TDP-43 2
pH	7.38 ± 0.02	7.38 ± 0.01	6.95	7.34	7.18	7.31
*p*O_2_ (mmHg)	74 ± 3.9	77 ± 9.5	61	69	43	44
*s*O_2_ (%)	94.25 ± 1.26	94.33 ± 1.75	78	92	65	74

Data for 4 GFP rats, 6 vehicle rats, 2 FUS rats, and 2 TDP-43 rats

### FUS and GFP transgene expression

**Fig. 4:**
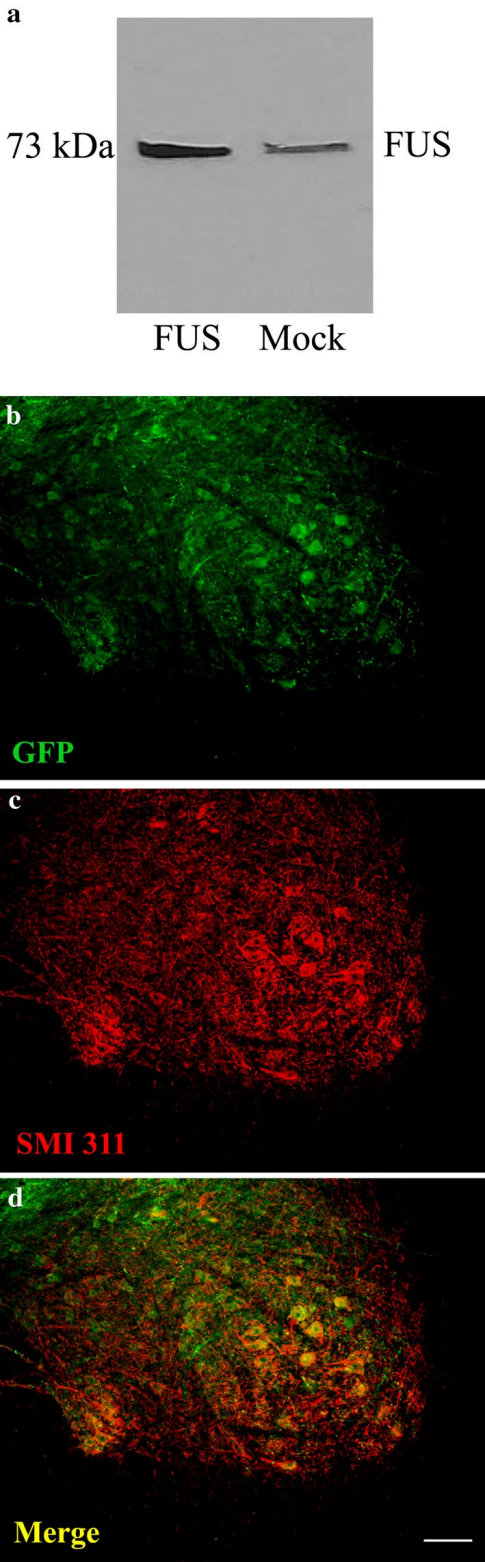
FUS and GFP transgene expression. **a** Transfection of the FUS DNA construct resulted in FUS overexpression in HEK293T cells at the expected molecular weight. **b** AAV9 GFP expression in the lumbar spinal cord of a rat that received AAV9 GFP 8 weeks earlier. GFP immunofluorescent staining is in **b**, SMI 311, a motor neuron marker antibody immunofluorescence is in **c**, and the merger is in **d**. The co-localization indicates GFP expression in motor neurons. *Bar* in **d** is 134 μm

We confirmed expression of FUS from the FUS DNA construct in transfected cells (Fig. 4a). The construct expresses an untagged form of human FUS. Using a FUS antibody that recognizes both rat and human FUS, we were unable to clearly detect up-regulated FUS expression in western blot samples of spinal cord, nor did we observe clearly increased immunohistochemical staining in the FUS animals. Thus, we can conclude that the level of FUS overexpression was subtle, below a several fold overexpression. We know that less than two-fold overexpression of TDP-43 in the spinal cord is sufficient to induce paralysis [37], so it is not surprising that low level FUS overexpression could induce the phenotype.

We have previously characterized the transgene expression pattern from AAV9 gene delivery to adult rats with this promoter construct, which incorporates the cytomegalovirus/chicken beta-actin promoter and the woodchuck hepatitis virus post-transcriptional regulatory element (WPRE) [27]. In the CNS, we can expect relatively robust expression in the spinal motor neurons, cerebellar Purkinje cells, and dorsal root ganglia neurons. The transgenes are also expressed in the liver, heart, and muscle in this system. We believe that expression in the spinal cord is essential for the motor deficits because we have experimented with a promoter that expresses TDP-43 in liver but not the spinal cord, which does not produce the paralysis (unpublished). Furthermore, we know that if we apply AAV9 TDP-43 (with the the hybrid cytomegalovirus/chicken β-actin (CBA) promoter as in this study) into the cerebral ventricles, we observe motor paralysis (unpublished), so we believe the motor deficits are mediated in the CNS. In contrast to FUS, the GFP in the AAV9 GFP group was easier to detect (Fig. 4b–d), presumably due to the lack of the endogenous protein and better signal-to-noise ratio. GFP was expressed in lumbar spinal motor neurons. There was co-localization of GFP and SMI 311 immunoreactivity (non-phosphorylated neurofilament), which labels motor neurons (Fig. 4b–d).

## Discussion

These data establish a relevant and consistent disease modeling system in rats based on intravenous FUS gene transfer for the first time. FUS exerted disease relevant symptoms with an adult onset, at a small fold-overexpression (less than twofold) in the spinal cord. This adult model is relevant to adult onset diseases, like ALS, and provides an experimental system that is far more amenable to pre-treatment baseline data collection as well as different interventional schedules compared to modeling systems in which the FUS expression begins earlier in development [33]. The FUS gene transfer produced progressive paralysis and decreased grip strength in adult rats. Plethysmographic analyses revealed rapid, shallow breathing in the FUS rats at the end stage of the disease state which was characterized by dramatic increases in respiratory frequency coupled with significant decreases in tidal volume. Although minute ventilation is the product of respiratory frequency and tidal volume, significant minute ventilation changes were observed due to the degree of increase in respiratory frequency. This breathing pattern can lead to hypercapnia due to ineffective gas exchange related to increased dead space. Consistent with the theory of inefficient gas exchange, there was arterial blood acidosis and decreases in *p*O_2_ and O_2_ saturation indicative of either pulmonary or diaphragmatic dysfunction. However, it is also possible that the hypoxia could have precipitated the rapid breathing. Hyperventilatory responses to hypoxia are mediated by chemoreceptor reflexes of oxygen-sensing cells in the carotid bodies which activate peripheral axons of petrosal ganglion neurons [38, 39]. These petrosal neurons project to the nucleus of the solitary tract in the brainstem [40, 41] which integrates and transmits afferent chemosensory activity to respiratory neurons that regulate respiratory rate and volume. In addition to affecting spinal cord motor neurons which innervate the diaphragm, there are a few cases suggesting that ALS-related neurodegeneration may also progress to involve brainstem nuclei that control the integration and processing of respiratory signals which could lead to deleterious breathing irregularities [42, 43].

We observed transgene expression in spinal motor neurons in this study, but we can only hypothesize that FUS expression in spinal motor neurons is what underlies the breathing changes. We attempted to evaluate phrenic motor neuron survival at the C4 region of the spinal cord to determine if the respiratory abnormalities seen were mediated in part by a loss of phrenic motor neurons, but we were limited due to technical issues. In addition to motor neurons, the effect could be mediated in the respiratory centers in the medulla or at the diaphragm muscle. More refinement of the expression pattern by altering the promoter, the type of AAV, as well as the route of administration could better pinpoint the site of action. Engineered AAVs will be advantageous to achieve better transduction efficiencies and better targeting, for example, AAV-PHP.B [44].

We anticipated that the breathing measurements, due to their precise quantitative features, would provide a sensitive, early stage marker and detect subtle changes, but the breathing changes were severe and arose consistently after the onset of the limb paresis/paralysis. However, we were able to closely and quantitatively monitor the onset of end stage in the FUS animals using plethysmography, in essence pinpointing the final stage in progression to death. Motor and respiratory deficits induced by FUS are relevant to ALS, and plethysmography offers a precise physiological readout to study treatments that may either delay or block the progression to end stage or conversely accelerate the time to end stage.

Respiratory failure and complications is the most common cause of death in ALS, and respiratory ability is a predictive factor in survival [45–47]. Patients with ALS typically develop respiratory problems after the onset of paralysis, although in rare cases respiratory impairments can be the presenting symptom [48]. The FUS rats followed the typical human sequence with motor abnormalities developing earlier than the respiratory abnormalities which appeared at the end stage. It is worth noting that the rapid, shallow breathing seen in the FUS rats mimics reports of ALS patients with decreased tidal volumes and increased respiratory frequency [5]. Interestingly, some similar relevant changes in breathing were also found in mSOD1 transgenic mice such as decreased tidal volume, although the mSOD1 mice developed slower respiratory frequency [6]. Rats at end stage in this model showed blood acidosis which is relevant to both human ALS and the mSOD1 mouse model. Blood acidosis seen in ALS patients has been shown to be related to poorer prognosis [46], and transgenic mSOD1 mice show acidosis in the end and moribund stages of disease [49]. Drugs that inhibit carbonic anhydrase, which regulates blood pH, have been shown to accelerate disease progression in mSOD1 mice [49]. This indicates that acidosis is not only a biomarker but also a potential contributor to the progression of disease. Since blood pH was only evaluated at end stage, we were unable to determine if acidosis preceded the respiratory dysfunction. An interesting next step would be a long-term catheter for longitudinal blood pH and blood gas readings to test if the hypoxia precedes the rapid breathing. However, the catheter could certainly affect motor function [50].

## Conclusions

Adult rats administered AAV9 FUS develop a consistent and ALS-relevant disease state including progressive motor paralysis and respiratory dysfunction. Since both RNA-binding proteins FUS and TDP-43 caused similar changes in motor function [27] and respiratory function, we hypothesize that both proteins exert similar or partial overlapping dysfunctions in RNA metabolism [51] that underlie the disease states. Plethysmography provided a precise monitoring of progressive changes in breathing parameters in FUS rats that are relevant to ALS and other ALS models. The relevant and consistent motor and respiratory symptoms may provide a useful pre-clinical model for therapeutic development as well as for studying underlying disease processes.

## Methods

### AAV vector production

The vector cassette was composed of AAV serotype 2 inverted terminal repeats, the hybrid cytomegalovirus/chicken β-actin promotor, a GFP, FUS, or TDP-43 construct, the woodchuck hepatitis virus post-transcriptional regulatory element (WPRE), and the bovine growth hormone polyadenylation sequence [52]. The vector cassette was packaged into AAV9 using helper and capsid plasmids from the University of Pennsylvania [53, 54]. All vectors were prepared under the same laboratory conditions. Viral stocks were sterilized via Millipore Millex-GV syringe filter (Billerica, MA), titered by dot blot assay, aliquoted, and stored frozen until use.

### Animals

Six to seven-week old female Sprague–Dawley rats (Harlan, Indianapolis, IN) were administered either AAV9 FUS (n = 8) at a dose of 3 × 10^13^ vector genomes (vg)/kg, AAV9 TDP-43 (n = 4) at a dose of 3 × 10^13^ vg/kg, AAV9 GFP (n = 4) at a dose of 3 × 10^13^ vg/kg to control for the expression of a foreign transgene, or 200 μl lactated Ringer’s solution (n = 6) as a vehicle control. Two of the TDP-43 rats were simultaneously co-administered AAV9 GFP at a dose of 2 × 10^13^ vg/kg. Female rats were used for consistency of measurements and lower and more stable body weights. Lower weights allow for less viral vector to be administered on a per kg basis, and more stable body weights helped ensure consistency of motor and lung function analyses. Estrus cycle was not controlled for in this study although some reports have shown increased respiratory frequency in the diestrus phase [55]. For vector administration, viral vectors were diluted to 200 μl in lactated Ringer’s solution and drawn up in a 1 ml syringe attached to a 30 gauge needle. Rats were anesthetized with isoflurane and placed on a heating pad. Their tails were swabbed with alcohol to better visualize the tail veins, and vector or vehicle was slowly administered to the lateral tail vein. Rats were allowed to recover from anesthesia before being returned to their cage. Weight and general health were monitored weekly. All animal procedures followed protocols approved by the institutional Animal Care and Use Committee and National Institutes of Health Guide for the Care and Use of Laboratory Animals. End stage euthanasia criteria included body weight less than 87.5% of the expected value and signs of pain including hunched posture, lethargy, porphyrin staining, decreased grooming, visibly increased respiratory frequency, or vocalization when touched. Three of eight animals injected with the AAV9 FUS failed to manifest breathing abnormalities by 8 weeks and only showed partial motor deficits relative to the other five rats in this group. We believe the less severe phenotype in these three animals was due to technical variability and for that reason they were not included in the data analysis.

### Rotarod

Rotarod (Rota-rod/RS, Letica Scientific Instruments, Barcelona, Spain) was conducted before injections (baseline) and then weekly for seven weeks following the injections. The rats were allowed to acclimate to the room for 30 min before testing began. Testing consisted of measuring the amount of time the rats can remain walking on the rotarod before falling off (latency to fall). The rod accelerated from 4 to 40 revolutions per minute (RPM) over 2 min, and the average of three trials was taken for each animal at each time point.

### Locomotor behavior

Locomotor analyses were conducted using a photobeam activity monitoring system from Coulbourn Instruments (TruScan 2.0, Whitehall, PA). The rats were allowed to acclimate to a dark room for 30 min before testing began. Tests were conducted for 30 min in the dark. Distance traveled and rearing were analyzed at baseline and weekly thereafter.

### Grip strength

Forelimb grip strength was analyzed at week 4 post-injection using a wire mesh attached to a 1 kg hanging scale (American Weight Scales, Inc., Norcross, GA). The scale was zeroed in the horizontal direction, and the rat’s forelimbs were placed in the middle of the mesh. The rat was pulled by the tail until it released the mesh. The average of three trials was taken for each rat.

### Escape reflex

Weekly, animals were lifted briefly by the tail three times and extension of the hindlimbs was observed. Hindlimb dysfunction was recorded only if the hindlimbs were clenched on all three trials.

### Plethysmography

Unanesthetized Sprague–Dawley rats were placed in an airtight unrestrained, whole-body plethysmograph chamber (Buxco rat chamber with Halcyon technology, Data Sciences International, St. Paul, MN, USA) outfitted with a digital video camera for simultaneous behavioral monitoring. While in the chamber, rats breathed normoxic (21% O_2_) room air that was continually exchanged at a constant flow rate. A sensitive very low differential pressure transducer (Validyne DP45; Northridge, CA, USA) connected to the chamber converted pressure changes associated with inspiration and expiration into respiratory waveforms. Integrated temperature and humidity probes were used to monitor and compensate for changes in the ambient conditions of the chamber. For each experiment, rats were allowed to acclimate in the chamber for ~15 min or until behavior stabilized, and then respiratory activity and video were simultaneously recorded and subsequently analyzed using Ponemah software (Data Sciences International, St. Paul, MN, USA) to measure tidal volume (ml), minute ventilation (ml/min), respiratory frequency (breaths per minute), inspiratory time, expiratory time, and total respiratory time. Respiratory parameters were averaged from ≥5 measurements per recording session, obtained by sampling 15–20 consecutive breaths every 3 min for 15–30 min. Tidal volume and minute ventilation were normalized to the animal’s weight to compensate for potential differences due to animal size. Rats were first recorded prior to injection to obtain baseline measurements and then weekly thereafter to monitor changes in respiratory physiology during disease progression. To control for potential effects of circadian rhythms, all rats were recorded during the light cycle and the order in which the rats were tested was varied weekly.

### Arterial blood collection and analysis

Rats were anesthetized with thiobutabarbital sodium (100 mg/kg) injected intraperitoneally. The femoral artery was cannulated for arterial blood gases analysis. The syringe used to draw the blood was coated in heparin (1000 USP units/mL) to prevent blood clotting. Approximately 0.3 ml of arterial blood from the femoral artery was removed and analyzed with an ABL5 blood gas analyzer (Radiometer Copenhagen, Brea, CA). Rats were euthanized immediately after arterial blood collection.

### Western blot

HEK293T cells were transfected with 2 μg of the FUS DNA construct or 2 μg of a DNA construct without a transgene via the calcium chloride method. Cells were collected after two days. The samples were sonicated in radioimmunoprecipitation assay (RIPA) buffer [1% nonidet-P40, 0.5% sodium deoxycholate, 0.1% sodium dodecyl sulfate (SDS), phosphate buffered saline (PBS)] with ethylenediamine tetraacetic acid (EDTA) and protease inhibitors (Pierce, Rockford, IL) and centrifuged. The supernatant was collected and protein content was determined by Bio-Rad Protein Assay Dye. Samples were normalized for protein content and electrophoresed in a 4–12% gradient polyacrylamide gel containing SDS (Bio-Rad). The primary antibody was anti-FUS (Abcam, Cambridge, MA), the goat anti-mouse secondary antibody was from Santa Cruz (Dallas, TX), and ECL reagents were from Amersham (Buckinghamshire, UK).

### Immunofluorescent staining

While anesthetized, animals were euthanized, and the spinal cord was dissected and post-fixed in 4% paraformaldehyde overnight at 4°C. Tissues were then transferred to 30% sucrose for cryopreservation. 50 μm sections of the spinal cord were cut using a sliding microtome with a freezing stage. Primary antibodies included anti-GFP (Invitrogen) and SMI 311 monoclonal antibody (Covance) at dilutions of 1:250 and 1:2000, respectively. Secondary antibodies included Alexa Fluor 488 and Alexa Fluor 594 (Invitrogen) at dilutions of 1:300.

### Statistical analyses

Statistical analyses were performed on GraphPad Prism 5.0. Statistical tests included two-way RM ANOVA with Bonferroni multiple comparison post-tests, one-way ANOVA with Bonferroni multiple comparison post-tests, and Mantel-Cox log-rank test as indicated. For all two-way RM ANOVAs, the within-subject factor (repeated measure) is time and the between subject factor is vector treatment. Data are expressed as mean ± standard error of the mean (SEM).

## Abbreviations

AAV: adeno-associated virus
AAV9: adeno-associated virus vector serotype 9
ALS: amyotrophic lateral sclerosis
ANOVA: analysis of variance
Bpm: breaths per minute
CBA: hybrid cytomegalovirus/chicken β-actin
CNS: central nervous system
EDTA: ethylenediamine tetraacetic acid
EWSR1: Ewing’s sarcoma breakpoint region 1
FUS: fused in sarcoma/translocated in liposarcoma
FTLD: frontotemporal lobar degeneration
FTLD-FUS: frontotemporal lobar degeneration with FUS-positive inclusions
GFP: green fluorescent protein
mSOD1: mutant form of copper, zinc superoxide dismutase 1
PBS: phosphate buffered saline
*p*CO_2_: partial pressure of carbon dioxide
*p*O_2_: partial pressure of oxygen
RIPA: radioimmunoprecipitation assay
RPM: revolutions per minute
RM ANOVA: repeated measures analysis of variance
SDS: sodium dodecyl sulfate
SEM: standard error of the mean
TAF15: TATA-binding protein associated factor 15
TDP-43: transactive response DNA-binding protein of 43 kDa
WPRE: woodchuck hepatitis virus post-transcriptional regulatory element
